# A Morphometric and Karyological Study of the *Anthemis macedonica* Group (Asteraceae, Anthemideae) Reveals a New Species from Greece

**DOI:** 10.3390/plants11213006

**Published:** 2022-11-07

**Authors:** Katerina Goula, Konstantinos Touloumis, Panayotis Dimopoulos, Theophanis Constantinidis

**Affiliations:** 1 Section of Ecology and Systematics, Department of Biology, National & Kapodistrian University of Athens, Panepistimiopolis, 15784 Athens, Greece; 2 Hellenic Agricultural Organization-DIMITRA, Fisheries Research Institute, Nea Peramos, 64007 Kavala, Greece; 3 Laboratory of Botany, Division of Plant Biology, Department of Biology, University of Patras, 26504 Patras, Greece

**Keywords:** *Anthemis*, Asteraceae, new species, karyology, morphometry, systematics, ultramafic substrates

## Abstract

A recent study of the *Anthemis* collections in the Balkans indicated that the taxa of the *Anthemis macedonica* group (*A. macedonica* subsp. *macedonica*, *A. macedonica* subsp. *thracica*, *A. meteorica*, *A. orbelica*) exhibit noteworthy morphological patterns not evaluated before. We applied morphometric approaches (principal components analysis, PCA; factor analysis on mixed data, FAMD) by considering 19 qualitative and 20 quantitative morphological characters, together with three ratios, in 26 populations of this group. Furthermore, the chromosome numbers and karyotype morphology were investigated in eight populations of the group, covering the taxa participating in the study. Our results revealed that the southernmost populations of the group represent a hitherto unknown species confined to serpentine: it is described here as *Anthemis serpentinica* Goula & Constantinidis. The morphological evidence supports the proximity of *A. macedonica* and *A. orbelica*, which would be better considered as subspecific entities of the same species. On the contrary, A. *meteorica* and *A. thracica* are retained as independent entities at species level. All taxa share the same diploid chromosome number of 2*n* = 2*x* = 18 with similar but not identical karyotypes. A brief description of all taxa, based on recent new collections, and a dichotomous key are presented. Lectotypes are designated for *Anthemis macedonica* and *A. meteorica*.

## 1. Introduction

*Anthemis* L., the second largest genus of Asteraceae tribe Anthemideae, comprises about 175 species in its narrow circumscription [1], and has a rather complex taxonomic and phylogenetic history. According to Lo Presti et al. [2], the pronounced variability of micro-morphological characters in *Anthemis* and the associated difficulty in recognising unique morphological features that consistently discriminate independent taxa, result in problems of its infrageneric classification. Several characters that were once used to define sections or even species within the genus, are proved to be unsatisfactory and of limited use. Oberprieler [3,4], for example, indicated that the distinction of sections composed of either perennials or annuals within *Anthemis* is problematic. Likewise, the presence or absence of scales on the receptacle disc does not necessarily characterise different species [1], whereas the appendages on the corolla lobes of disc florets “are too variable to characterise species or species groups” [5].

The group of taxa around *Anthemis macedonica* Boiss. & Orph. is not an exception to the above-cited rule. According to Dimopoulos et al. [6], there are three representatives of this group in Greece: *Anthemis macedonica* subsp. *macedonica*, *A. macedonica* subsp. *thracica* (Griseb) Oberpr. & Greuter and *A. orbelica* Pančić. The related *A. macedonica* subsp. *stribrnyi* (Velen.) Oberpr. & Greuter occurs in Bulgaria. All four taxa are endemic to the Balkan Peninsula [7] and each one has suffered a taxonomic and nomenclatural odyssey [8,9,10,11,12,13,14,15,16,17,18,19,20,21]. By the beginning of the 20th century *A. orbelica* itself had been described no less than five times, under five different names, all based on plants of the Rila Mountain in Bulgaria: *A. macedonica* Pančić [11], *A. orbelica* Pančić [12], *A. halacsyi* Formánek [13], *A. orbelica* Velenovský [14] and *A. riloensis* Velenovský [15]. By 1903, there were already so many and contradictory descriptions of its morphology that led Velenovský [15] to speak for “an embarrassing situation” and accuse his colleagues of “unclear, inadequate and wrong diagnoses”.

Τhe sectional placement of *Anthemis macedonica* and its allies needs further elucidation. Fernandes [20] indicated that plant longevity or lifespan seems to be important in ordering the species into sections. *A*. *macedonica* subsp. *macedonica*, an annual to biennial species [9,19,20], is considered a member of section *Anthemis*, a group that covers most of the annual species of *Anthemis* s.str. (i.e., excluding *Cota*). *A. orbelica* on the pther hand, a biennial to perennial species [11,12,13,14,19,20], is considered a member of section *Hiorthia* (DC.) R. Fernandes, a group that includes the perennial species of the diverse *A. cretica* L. group. The sectional placement of *A. macedonica* subsp. *thracica* has not been clarified; however, most authors considered it as a biennial or perennial [8,19]. Molecular phylogenetic analyses confirmed the placement of *A. orbelica* into section *Hiorthia* [2,3]. Lo Presti et al. [2] showed that *A. macedonica* is closely related to *A. orbelica*, as they form, together with *A. rumelica* (Velen.) Stoj. & Acht. and *A. hydruntina* E. Groves, a separate subclade within the *A. cretica* clade, in a Bayesian Inference tree of nrDNA ITS data. This close relation had already been noticed by Greuter et al. [21], who combined *A. orbelica* as a subspecies of *A. macedonica*: *A. macedonica* subsp. *orbelica* (Pančić) Oberpr. & Greuter.

According to Fernandes [20], the diagnostic characters that keep the *Anthemis macedonica* group members coherent, despite their varying lifespan, are the very narrow leaf-lobes (less than 1 mm), the presence of numerous sessile glands in all parts of the plant, the quadrangular inner achenes and the granulate to tuberculate surface of at least the outer achenes. Still, some of these characters can be observed independently in other *Anthemis* species as well, mainly in members of section *Hiorthia*, while some show a great variability within *A. macedonica* and even overlap between the different taxa of this group.

This study aims to delve into the diversity of the *Anthemis macedonica* group, particularly in the southern part of its distribution. It is also an attempt to define the characters that delimit the members of the group and to consider entities closely related to the group. For this reason, we also included *Anthemis meteorica* Hausskn. ex Nyman in the present study. This is a somewhat puzzling species originally described from the Meteora area (Central Greece, see [22]), but later merged into the synonymy of *A. cretica* [6]. The latter name includes a diverse group of strictly perennial members with numerous non-flowering shoots during the flowering period, and usually unbranched flowering stems [20,23]. *A. meteorica*, on the other hand, although described as a perennial species, shows the same instability in its lifespan observed within members of the *A. macedonica* group, and moreover, presents the combination of diagnostic features used by Fernandes [20] to circumscribe this particular group.

While collecting material for the study of the *A. macedonica* group, we realised that some populations from Central Greece (*Anthemis 1*) should be included in this study for the same reasons as *A. meteorica*. These populations exhibit certain morphological deviations worthy of careful evaluation. Unlike most members of the *A. macedonica* group, they are restricted to an area of ultramafic bedrock, a particular rock type rich in Μg, Fe and Si, which in our case also contains high concentrations of heavy metals, such as Cr, Cu and Mn [24]. The ultramafic substrates quite often include endemic species adapted to their geoedaphics [25].

## 2. Results

### 2.1. Morphometric Analyses

**Figure 1:**
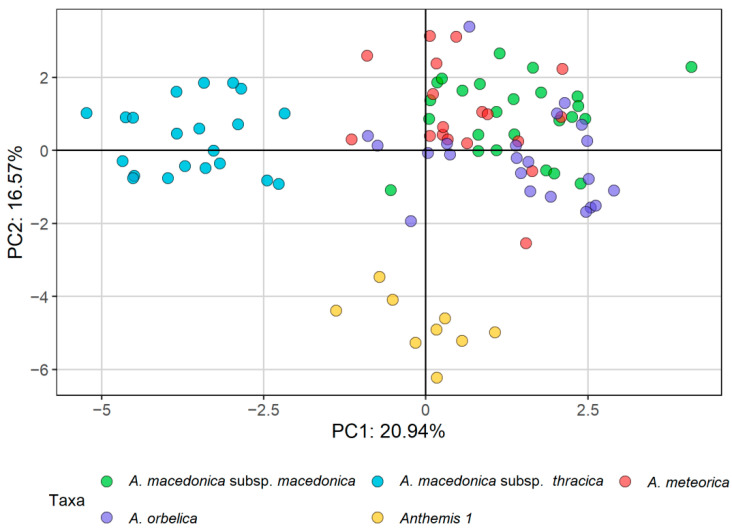
Principal component analysis (PCA) of the *Anthemis macedonica* group, based on 20 quantitative characters and three ratios.

**Figure 2:**
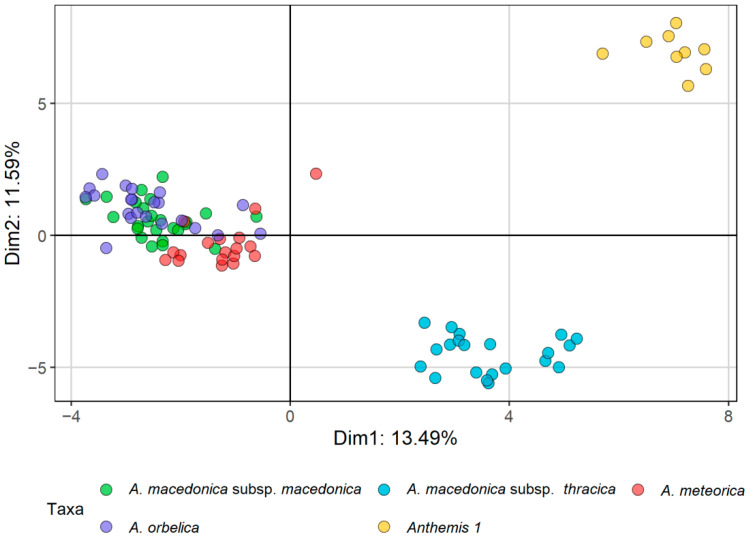
Factor analysis (FAMD) of the *Anthemis macedonica* group, based on 20 quantitative characters, three ratios and 19 qualitative characters.

Τhe morphometric analyses that we used (principal components analysis, PCA; factor analysis on mixed data, FAMD; Figure 1 and Figure 2, respectively) indicated a clear distinction of *Anthemis macedonica* subsp. *thracica* and *Anthemis 1* from the rest of the examined material, i.e., *A. macedonica* subsp. *macedonica*, *A. meteorica* and *A. orbelica*. This distinction was more evident in the FAMD, i.e., when evaluating both the qualitative and quantitative characters (Figure 2). In the same analysis, the group of *A. meteorica* was also defined, albeit remaining close to the *macedonica* subsp. *macedonica*–*A. orbelica* complex, which appeared coherent.

**Figure 3:**
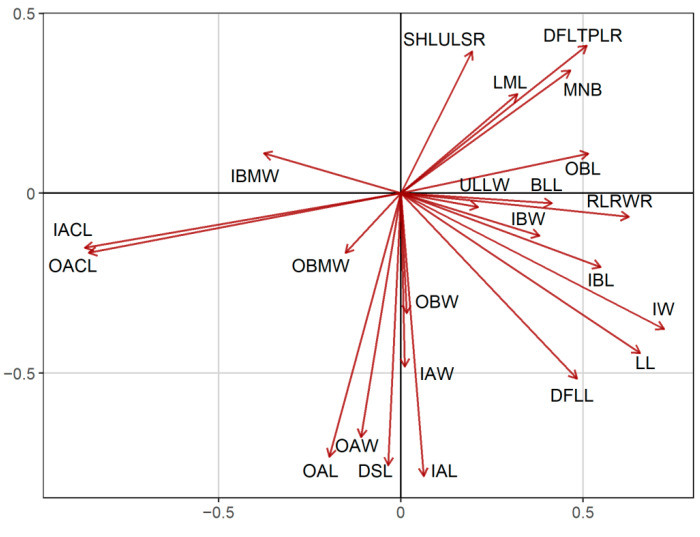
Relative contribution of the morphological characters to the PCA of Figure 1.

**Figure 4:**
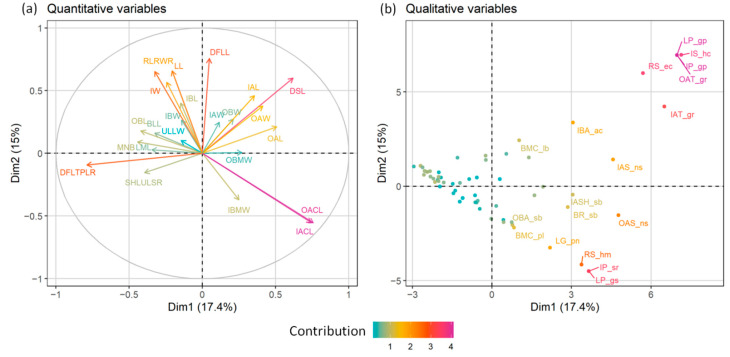
Relative contribution of the quantitative (**a**) and qualitative (**b**) morphological characters to the FA of Figure 2.

In both the PCA and FAMD, the quantitative characters that had the most significant relative contribution to the ordination of individuals (Figure 3 and Figure 4a) were mainly those referring to the achene size and the corona length (OACL, IACL, see Table 1). The number of stem leaves divisions (DSLs), as well as the involucre width (IW) and the size of both the ligules and disc florets (LL, DFLL, DFLTPLR), also contributed significantly to the analysis. Regarding qualitative characters, the surface of the disc floret achenes (OAT, IAT), the pubescence of the involucre and the leaves (IP, LP), together with the shape of the involucre and the receptacle were among those that participated the most to the ordination of the FAMD (Figure 4b). *Anthemis 1*, emerging as the most distinct group of individuals in both analyses, split off from the rest of the groups because of its larger disc floret achenes (OAL, IAL, OAW, IAW), the more dissected leaves (DSLs), the tomentose leaves and involucre (LP, IP), the hemispherical to obconical shape of the involucre (IS) and the elongated-conical, sharply acute receptacle (RS) (Figure 3 and Figure 4). *A. macedonica* subsp. *thracica* was defined as another distinct group, separated by the longer coronas in both the outer and inner disc floret achenes (OACL, IACL), the dense, sericeous pubescence on the leaves and involucre (LP, IP) and the shorter, hemispherical to conical receptacle (RS) (Figure 3 and Figure 4).

**Figure 5:**
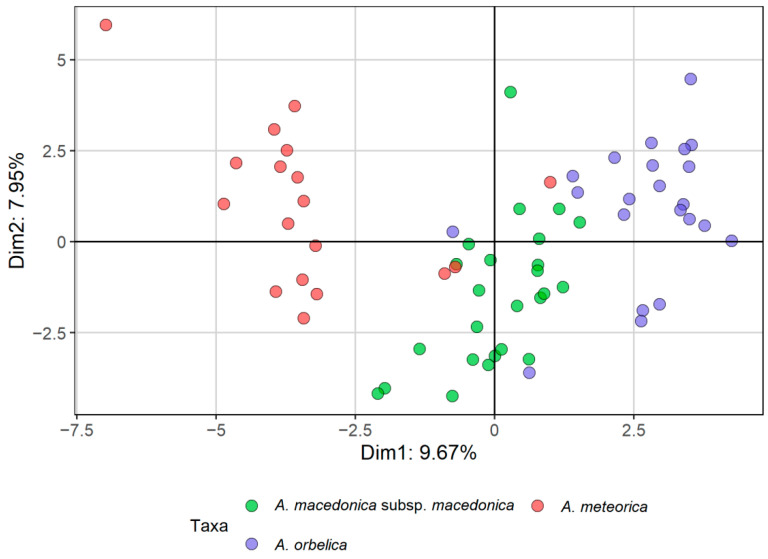
Factor analysis (FAMD) of *Anthemis macedonica* subsp. *macedonica*, *A. meteorica* and *A. orbelica*, based on 20 quantitative characters, three ratios and 19 qualitative characters.

**Figure 6:**
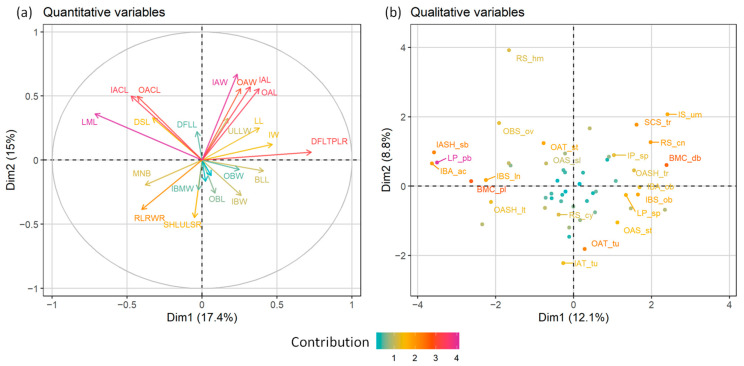
Relative contribution of the quantitative (**a**) and qualitative (**b**) morphological characters to the FAMD of Figure 5.

A second FAMD was attempted after excluding the well-circumscribed *Anthemis 1* and *A. macedonica* subsp. *thracica* from the group. The results, as well as the contribution of both the qualitative and quantitative characters to this analysis are shown in Figure 5 and Figure 6a,b. *A. meteorica* formed a well-defined group, compared to the remaining two taxa, a conclusion that strengthens the results shown in Figure 2. The variables that have the most significant contribution to this FAMD are the leaf pubescence (LP), the characters of the bracts likemargin colour and shape (BMC, IBS, IBA), the ratio of the total disc floret length to the length of the swollen part (DFLTPLR), and the leaf mucro length (LML) (Figure 6). *A. meteorica* is detectable from the rest of the individuals, mainly by its more acuminate leaf-lobes (LMLs), its pubescent leaves (LP), the more acute apex of its inner bracts, compared to the obtuse to sometimes subacute apex of the two remaining taxa (IBA), and by the characters of the receptacle, reflected both in the quantitative ratio of the receptacle length to width (RLRWR) and the qualitative receptacle shape (RS) (Figure 6). The slightly longer and wider disc floret achenes (IAW, IAL, OAW, OAL) and the darker bracts (BMC), as well as the regularly cupuliform involucre (IS) and the conical receptacle furnished with scales, often trifid at the apex (RS, SCS) that characterise *A. orbelica*, are the most reliable characters that support its distinction from *A. macedonica* subsp. *macedonica* (Figure 6).

### 2.2. Karyological Analyses

**Figure 7:**
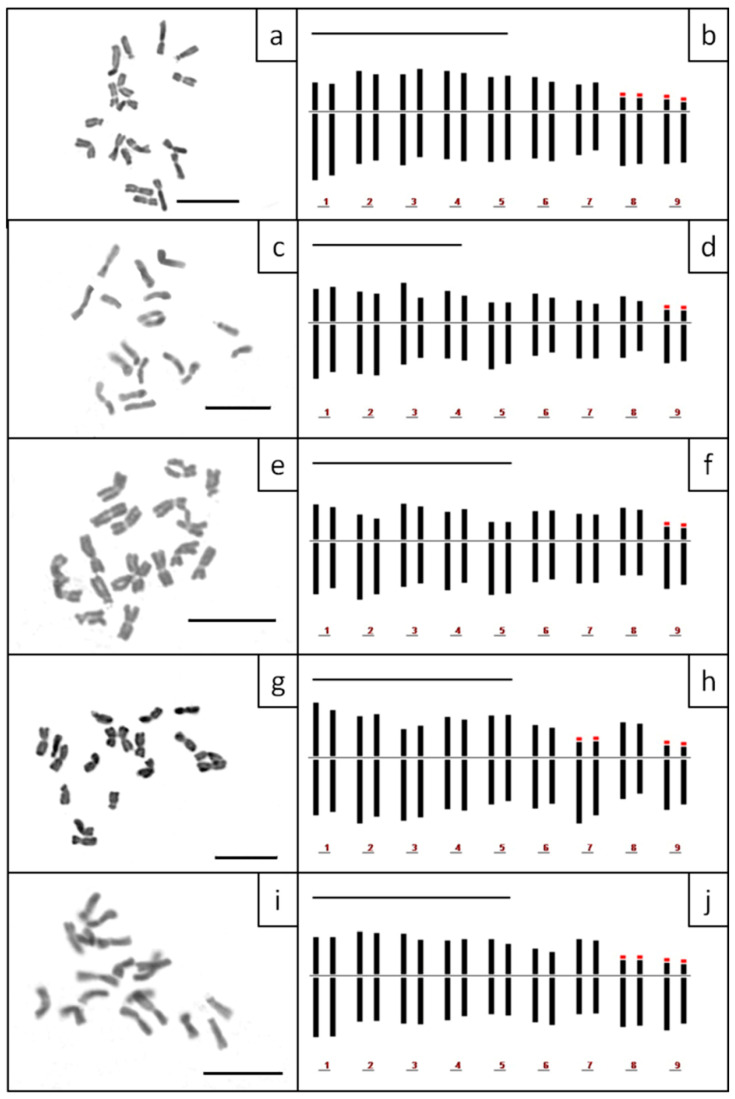
Metaphase plates and idiograms of *Anthemis macedonica* subsp. *macedonica* (**a**,**b**), *A. macedonica* subsp. *thracica* (**c**,**d**), *A. meteorica* (**e**,**f**), *A. orbelica* (**g**,**h**) and *Anthemis 1* from the ultramafic areas of Central Greece (**i**,**j**). Red parts of the idiograms indicate satellites. Scale bars = 10 μm.

All populations of the *Anthemis macedonica* group examined, share the same diploid chromosome number of 2*n* = 2*x* = 18 (Table 2). Diploid populations of *Anthemis macedonica* subsp. *macedonica*, *A. macedonica* subsp. *stribrnyi* and *A. orbelica* are known to grow in Bulgaria [26]. Aneuploidies have also been reported in *A. orbelica* and *A. macedonica* subsp. *stribrnyi* (2*n* = 18 + 4 and 2*n* = 18 + 3, respectively) together with a triploid population (2*n* = 3*x* = 27) of *A. orbelica* from Mt Rila [26]. The chromosome numbers, metaphase plates and idiograms of *A. macedonica* subsp. *thracica*, *A. meteorica* and the population from the serpentine area of Central Greece (*Anthemis 1*) are presented here for the first time (Figure 7).

**Figure 8:**
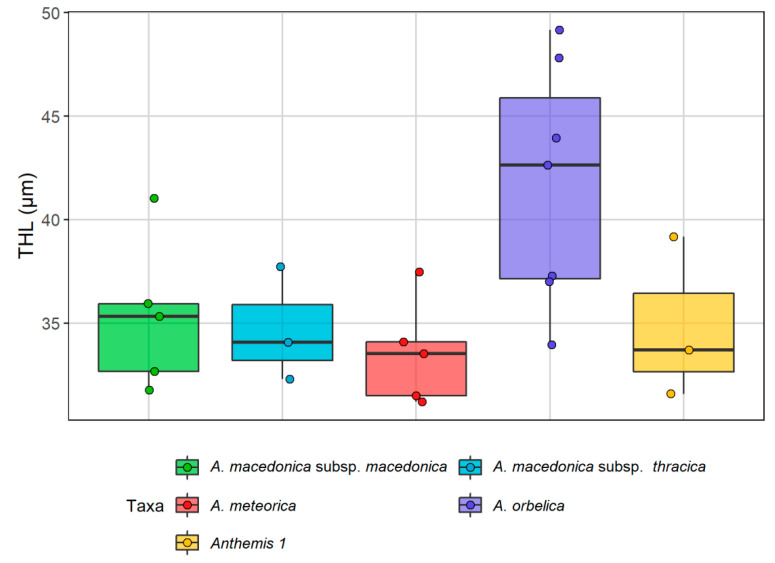
Box plots presenting the variation of the total haploid length (THL) in five different taxa of the *Anthemis macedonica* group. The coloured points present the individuals’ values for each taxon.

**Figure 9:**
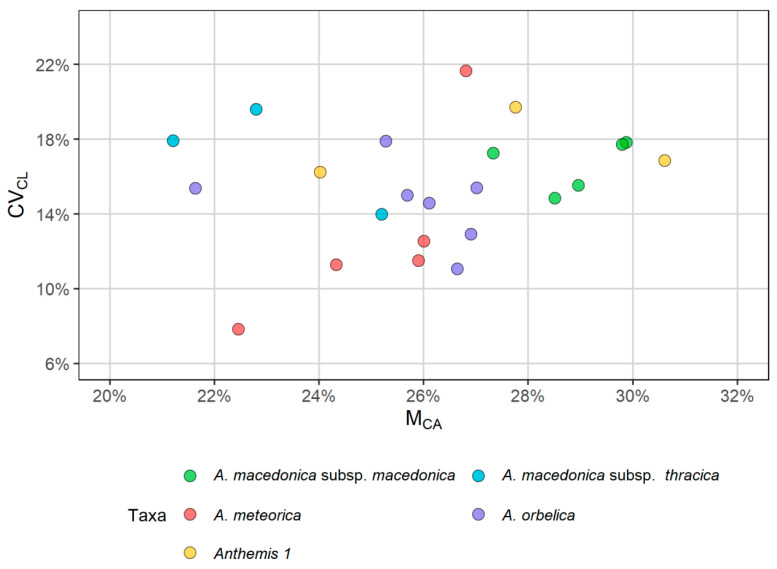
Scatter plot showing the karyotype indices of the intrachromosomal (M_CA_) and interchromosomal (CV_CL_) asymmetry for all the studied taxa in the *Anthemis macedonica* group.

With respect to the karyotype formula, all populations had 12 metacentric (m) chromosomes (Table 2), which appeared very similar amongst the taxa. However, there was an interesting differentiation concerning the remaining submetacentric (sm) and subtelocentric (st) chromosomes: two sm and four st chromosomes were present in all metaphase plates of *A. macedonica* subsp. *macedonica*, *A. orbelica* and the populations from the serpentine area of Central Greece, whereas four sm and two st chromosomes were present in all metaphase plates of *A. meteorica* and *A. thracica*. Small satellites were observed on the short arm of the st chromosomes in all cases, whilst an additional satellited sm chromosome pair was observed in a metaphase plate of *A. meteorica*. The lack of satellites on the sm chromosomes in the rest of the *A. meteorica* metaphase plates, as well as the observed inequality of certain chromosome pairs in the ideogram reconstructions, may partly be an artifact of image processing. THL varies from 31.19 to 49.16 μm. *A. orbelica* tend to have a longer THL, compared to the rest of the taxa (Figure 8), although there are not enough data to test the statistical significance. A scatter plot of the asymmetry indices M_CA_ [27] and CV_CL_ [28] constructed by 21 metaphase plates did not contribute any further to the distinction of the different taxa, based on the chromosome features (Figure 9). The same result is reached with different asymmetry indices (CV_CI_–CV_CL_ [28]; A1–A2 [29]).

## 3. Discussion

### 3.1. Evaluation of Taxa within the Anthemis macedonica Group

Our investigation aimed to shed light on the variable morphological complex of *Anthemis macedonica*, a group of taxa that have been treated in different ways and at various taxonomic levels in the past. Although closely related, from a phenetic and karyological point of view, some of these taxa had been allocated to different *Anthemis* sections, thus hampering the assessment of their actual relationships. Our study was particularly concentrated in the southern parts of the complex distribution, where certain populations clearly did not fit the known morphological patterns of the group.

**Figure 10:**
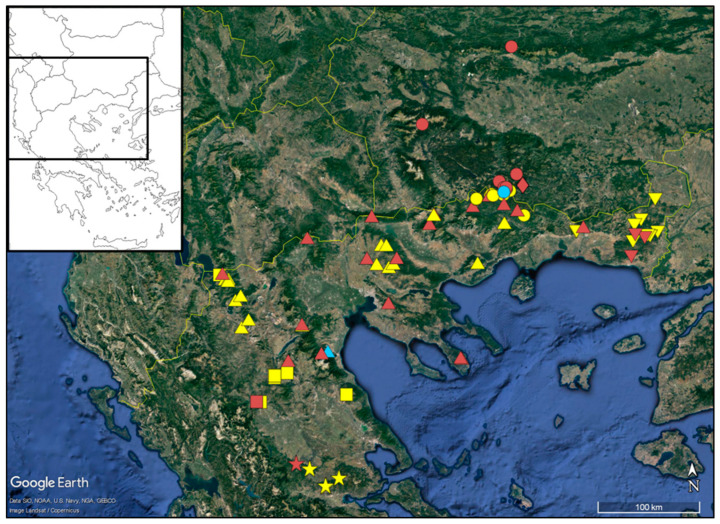
Distribution map of *Anthemis macedonica* group, showing the localities of the specimens collected by the authors (yellow and sky-blue colour) and those of the examined herbarium specimens (red colour). Triangles: *A. macedonica* subsp. *macedonica*; circles: *A. macedonica* subsp. *orbelica*; squares: *A. meteorica*; stars: *A. serpentinica*; reversed triangles: *A. thracica*; rhombus: *A. macedonica* subsp. *stribrnyi*; sky-blue triangle: possible hybrid between *A. macedonica* subsp. *macedonica* and *A. meteorica*; sky-blue circle: possible hybrid between *A. macedonica* subsp. *orbelica* and *A. pindicola*. (Background map data: Google, SIO, NOAA, U.S. Navy, NGA, GEBCO.)

The morphometric and chromosome data support the placement of *Anthemis macedonica* s.str. and *A. orbelica* under a single taxonomic entity. Greuter et al. [21] expressed the same opinion, based on morphological grounds and Lo Presti et al. [2] provided some evidence of phylogenetic proximity. Kuzmanov et al. [26] corroborated, based on their karyotype similarity, and argued that these taxa, at subspecific level, namely *A. macedonica* subsp. *macedonica* and *A. macedonica* subsp. *orbelica*, should be members of sect. *Hiorthia*, thus contradicting Fernandes [20]. Although these two taxa appear to have predominately—but not always—a different life cycle and some fine morphological differences (Table 3, Key to taxa), they are otherwise difficult to distinguish. Their habit, and in particular their lifespan and overall size, seem to be dependent also on environmental factors. In our results (Figure 6b) longevity (or otherwise, lifespan) has an insignificant contribution to the ordination of individuals that belong either to *A. macedonica* subsp. *macedonica*, or *A. macedonica* subsp. *orbelica* and *A. meteorica*. Field observations indicated that plants from high montane habitats that mostly grow in moist forests (Mt Rodopi, Mt Vitsi) keep quite often a biennial or short-lived perennial life-form, with a few well-developed leaf rosettes at flowering time. On the other hand, plants from drier habitats at lower altitudes are usually annuals, without additional leaf-rosettes at flowering time. This observation comes in agreement with the life-form shifts deduced in *Anthemis*, where the annual habit appeared to have evolved several times and independently in various groups during the past, following a progressive aridification in the Mediterranean area [1]. *A. macedonica* subsp. *macedonica* and subsp. *orbelica* meet in Greece, close to the borders with Bulgaria, but show a parapatric distribution around the Mt. Rodopi area (Figure 10).

Field observations and cultivation experiments regarding *Anthemis meteorica,* revealed that this species has a variable lifespan shared with the members of the *A. macedonica* group, rather than those of the *A. cretica* group. In its habitat, it mostly behaves as a biennial or a short-lived perennial, but some annual plants were observed as well. Its close affinity to *A. macedonica* became obvious in our morphometric analyses: the representatives of *A. meteorica* form a coherent group together with *A. macedonica* subsp. *macedonica* and subsp. *orbelica* (Figure 1 and Figure 2). However, when samples of all three taxa were analysed as a single entity, *A. meteorica* appeared to stand out (Figure 5). Therefore, we treat *A. meteorica* as an independent species with affinities to the *A. macedonica* group. The shape and colour of the involucral bracts are useful discriminating features between these two taxa. In *A. meteorica*, all involucral bracts have a very thin margin, concolorous to the remaining bract part, and a prominent, light green midvein, whereas *A. macedonica* s.l. usually has a brown margin and a green midvein, very distinct from the rest of the bract. Furthermore, the inner involucral bracts in *A. meteorica* are more lanceolate and acute than those of *A. macedonica* (Table 3). Some significant differences were also found in the karyotypes of these two species (see Results and Table 2) indicating that, despite their overall morphological similarity, *A. meteorica* would better be treated as a separate species. Most of the *Anthemis meteorica* populations occur south of the *A. macedonica* distribution area (Figure 10) and we presume that new populations can be found to the south and to the west or north-west of the latter species.

*Anthemis macedonica* subsp. *thracica* was attributed to section *Hiorthia* since its very beginning. Grisebach [8] described it as a variety of *Anthemis montana* L., with the latter accepted nowadays as a synonym of *A. cretica*. A more recent work [19] considered it a separate species and placed it close to the *A. macedonica* group. This group was re-arranged by distinguishing three varieties under *A. thracica*: var. *orbelica* (Panč.) Stoj. et Acht., var. *macedonica* (Boiss. et Orph.) Stoj. et Acht., and var. *stribrnyi* (Vel.) Stoj. et Acht., which correspond to three out of the four currently [7] accepted subspecies of *A. macedonica*. In this work [19] *A. thracica* was regarded as a biennial species, thus adding a second life-form to its perennial habit provided by Grisebach [8]. According to our morphometrical and karyological results, *A. thracica* is well-distinguished from the *A. macedonica* complex and should be treated as a separate species (Figure 1 and Figure 2). *A. thracica* shares several features in common with *A. rumelica* [18,20]. However, they present obvious differences in the morphology of their disc floret achenes: the latter has achenes with a very short auricle, while the former has a relatively long corona, measuring 0.4–0.7 mm and reaching 1/3 of the achene’s body in the outer achenes, whereas the corona further increases to the 1/2 of the achene’s body in the inner achenes. The morphological features useful in distinguishing *A. thracica* from the rest of the group are given in Table 3 and in the Key to Taxa.

*Anthemis thracica* is distributed in East Macedonia and Thrace (Figure 10) and shared between Greece and Bulgaria. It appears to be allopatric with respect to the rest of the taxa in its group; however, its border area with *A. macedonica* should be better investigated for the possible discovery of new, neighbouring populations.

The *Anthemis 1* populations from the serpentine parts of Central Greece stands out in our results as a distinct *Anthemis* group well separated from the rest of the *A. macedonica* taxa (Figure 1 and Figure 2). They seem to have a stabilised biennial lifespan, documented by field observations carried out during different months of the year. In winter, particularly, we noticed no sign of last season’s living flowering stems on any plant, only leaf rosettes. Several plants with well-developed leaf rosettes but without flowering stems were also found intermixed with flowering individuals during late spring. The karyotype of this species is similar to that of *A. macedonica*, but several morphological differences, i.e., type of pubescence, bracts and achene shape and size, disc floret features and receptacle shape, allow for safe distinction between the two species (Table 3). The achenes of *Anthemis 1* resemble those of the *A. cretica* group; however, the strictly biennial lifespan and the sharp, elongated conical receptacle differentiate the species from *A. cretica* and bring it closer to *A. macedonica*.

The geological substrate of *Anthemis 1* is also unusual: it appears to be the only member of the *A. macedonica* group confined to ultramafic rock types, also known as ophiolites or serpentines, in Greece. The wider serpentine area of East Sterea Ellas is also home to a few local Greek endemics (*Onosma stridii* Teppner, *Polygonum papillosum* Hartvig) and hosts several Greek serpentine endemics with a wider distribution.

Based on the evidence presented above, we describe *Anthemis 1* as a new species, *Anthemis serpentinica* Goula & Constantinidis. For a detailed description of the species and further comments see under Taxonomic Treatment. Its currently known localities appear in the Appendix A.

### 3.2. Key to Taxa

1. Leaves and involucral bracts tomentose to sericeous ……………………………………2

– Leaves slightly pubescent to pubescent; involucral bracts almost glabrous to slightly pubescent ………………………………………………………………………………………... 3

2. Plants predominantly or obligatory biennial, at least some non-flowering rosettes present together with flowering plants in the field; receptacle elongated, conical, acute; disc florets 2.8–3.2 mm long; disc floret achenes with a corona not more than 0.3 mm long ……………………………………………………………………………………... *A. serpentinica*

– Plants predominantly perennial, sometimes flowering the first year; receptacle hemispherical to shortly conical; disc florets 2.2–2.3(–2.5) mm long; disc floret achenes with a conspicuous corona 0.4–0.7 mm long ………………………………………. *A. thracica*

3. Inner involucral bracts oblanceolate to obovate, apex usually obtuse; involucral bracts margin light to dark brown, their midvein green, distinct from the yellowish-green bract ……...……………………………………………………………………………………………... 4

– Inner involucral bracts lanceolate to oblanceolate, apex subobtuse to subacute; involucral bracts margin usually not coloured, their midvein prominent, but almost concolorous to the bract ……………………………………………………………. *A. meteorica*

4. Plants predominantly annual, sometimes biennial or short-lived perennial; leaf rosettes absent at flowering period; involucre almost glabrous, hemispherical; ultimate leaf lobes 0.4–0.7 mm wide …………………………………………….. *A. macedonica* subsp. *macedonica*

– Plants predominantly biennial, sometimes annual or short-lived perennial; leaf rosettes usually present at flowering period; involucre almost glabrous to slightly pubescent, often cupuliform; ultimate leaf lobes 0.5–1 mm wide ………... *A. macedonica* subsp. *orbelica*

### 3.3. Taxonomic Treatment

**Figure 11:**
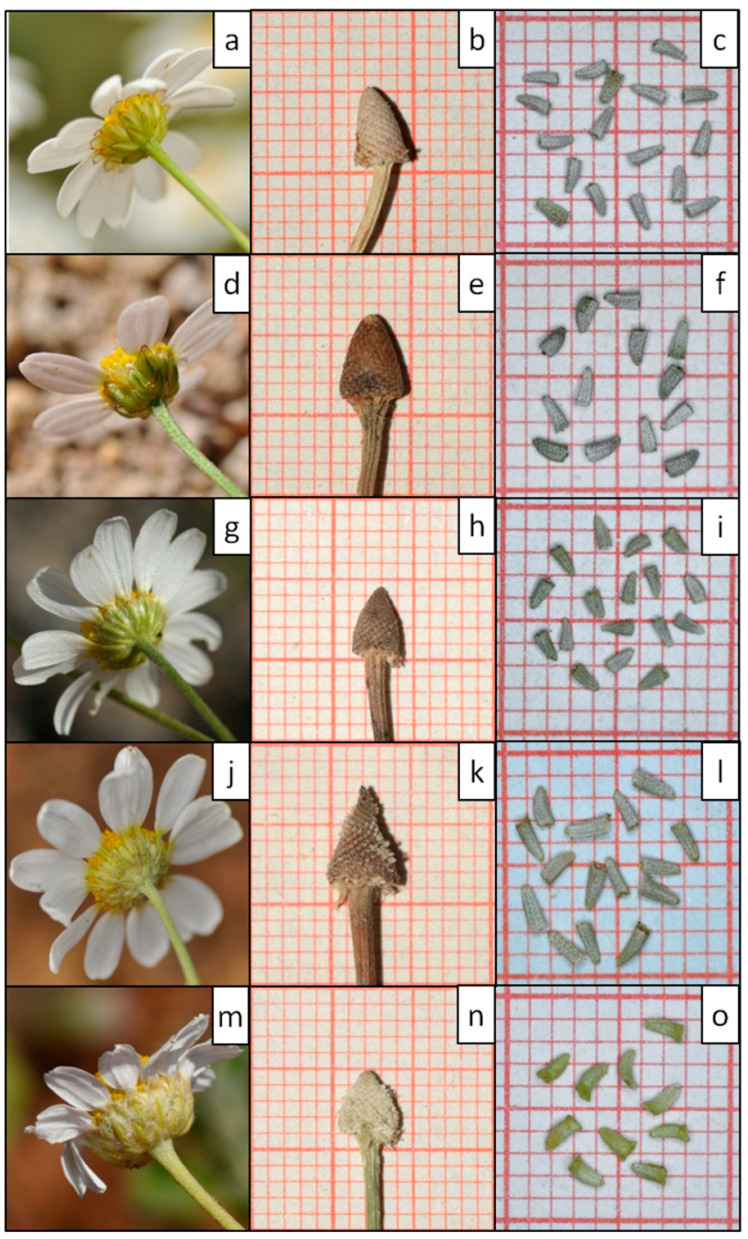
Comparative photographic material of the involucres (**a**,**d**,**g**,**j**,**m**), receptacles (**b**,**e**,**h**,**k**,**n**) and disc floret achenes (**c**,**f**,**i**,**l**,**o**) of the five taxa presented in this study: *Anthemis macedonica* subsp. *macedonica* (**a**,**b**,**c**), *A. macedonica* subsp. *orbelica* (**d**,**e**,**f**), *A. meteorica* (**g**,**h**,**i**), *A. serpentinica* (**j**,**k**,**l**) and *A. thracica* (**m**,**n**,**o**). Square sides = 1 mm. (Note: The d capitulum has an unusual low number of ray florets).

***Anthemis macedonica*** Boiss. & Orph. subsp. ***macedonica*** (1859: 97) (Figure 11)

*Lectotype (designated here)*: Greece. Legi in reg. super. montis Corfiati, Macedoniae, 20/7/1857, *Orphanidis 3614* (holotypeG00764108, G-BOIS!).

*Isolectotypes:* In regione superiori montis Korthiati, 20 July 1857, *Orphanidis 3614* (ATHU!); In m. Kothiati Macedoniae, 20/7/1857, *Orphanidis s.n.* (LY0017447!).

*Description:* Annual, biennial or sometimes short-lived perennial, glabrescent, more or less densely glandular-punctate in all parts. Indumentum of medifixed hairs, when present. Stems single or several, erect, 10–50 cm long, branched above the middle, striate, often subquadrangular and reddish-brown, at least in the lower parts. Non-flowering shoots usually absent at flowering. Leaves pinnatisect, the primary segments patent, sparsely pubescent to glabrous; ultimate leaf lobes very narrow, 0.4–0.7 mm wide, narrowly obovate-oblanceolate to linear, with a very short mucro (<0.1 mm). Lower stem leaves 1.5–4 cm, 2(-3)-pinnatisect; upper leaves smaller and less dissected, 1(-2)-pinnatisect. Peduncles (3–)5–10(–12) cm long, leafless, except for small, scale-like, entire leaves; capitula solitary. Involucre 8–10 mm wide, hemispherical, glabrescent; involucral bracts pale yellowish-green, with a darker green midvein, the margins light to dark brown; outer bracts 2–3.5 × 1–1.7 mm, lanceolate to oblanceolate, subacute to acute; inner bracts 3–6 × 1.3–2 mm, oblanceolate to obovate, usually obtuse. Ligules white, 8–11 mm. Disc florets yellow, (2.4–)2.5–2.8(–3) mm, lower 1–1.2 mm part swollen at maturity, lobes 0.3–0.4(–0.5) mm long. Receptacle shortly conical to hemispherical-cylindrical, 3–6 × 2.5–4 mm; receptacular scales oblong-obovate, tapering into a rigid median nerve ca. 0.5 mm long, about equaling disc-florets. Ray floret achenes tuberculate, striate, trigonous, curved, ca. 1.5 mm, with a rim ca. 0.1 mm. Outer disc floret achenes usually tuberculate and slightly striate, usually subquadrangular or trigonous, curved, 1.3–1.8 mm, with an acute rim ca. 0.1 mm, sometimes slightly oblique; inner disc floret achenes tuberculate to smooth, slightly striate, turbinate, subquadrangular, 1.6–1.8 mm, with an acute rim ca. 0.1 mm.

*Distribution and habitat:* Balkan endemic, distributed in Greece, Bulgaria, North Macedonia and Serbia [7]. In Greece, it is restricted to the northern part of the country. Rare in Serbia, known only from the southernmost parts of the country [30]. It grows on a variety of habitats, such as woodland-edges, open meadows and scrub, often in semi-shade, at the wide altitudinal range of 200–2000 m.

***Anthemis macedonica* Boiss. & Orph.** subsp. ***orbelica*** (Pančić) Oberpr. & Greuter (2003: 40) (Figure 11)

*Basionym: A. orbelica* Pančić (1886: 27).

*Type:* Bulgaria. Mt Rila, *Pančić s.n.* (holotype, BEOU 9939!, see https://pancic.bio.bg.ac.rs/Yu/Nomen/pages/007.html, accessed on 30 June 2022).

*Synonyms: A. halacsyi* Formánek (1898: 55); *A. orbelica* Velen. (1902: 155); *Anthemis riloensis* Velen. (1903: 6).

*Description:* Biennial or short-lived perennial, sometimes flowering the first year, sparsely hairy to glabrescent, more or less densely glandular-punctate in all parts. Indumentum of medifixed hairs, when present. Stems single or several, erect, (17–)25–65 cm long, branched above the middle, striate, often subquadrangular and reddish-brown, at least in the lower parts. Non-flowering shoots sometimes present at flowering. Leaves pinnatisect, the primary segments patent, usually sparsely pubescent to subglabrous; ultimate leaf lobes very narrow, 0.5–1 mm wide, narrowly obovate-oblanceolate to linear, with a very short mucro (<0.1 mm). Lower stem leaves 2–4 cm, 2-3-pinnatisect; upper leaves smaller and less dissected, 1(-2)-pinnatisect. Peduncles (5–)7–15 cm long, leafless, except for small, scale-like, entire leaves; capitula solitary. Involucre 8–11 mm wide, hemispherical to cupuliform, usually slightly pubescent to glabrescent; involucral bracts yellowish-green, with a darker green midvein, the margins usually brown to dark brown; outer bracts 2.2–3 × 1–1.8 mm, trigonous or ovate to lanceolate, subacute to acute; inner bracts (3–)4–5 × 1.2–2 mm, oblanceolate to obovate, obtuse to subacute. Ligules white, 7–14 mm. Disc florets yellow, (2.3–)2.5–2.8 mm, lower 1–1.1 mm part swollen at maturity, lobes 0.3 mm. Receptacle shortly conical to hemispherical-cylindrical, 2.5–5 × 2.5–4 mm; receptacular scales oblong-obovate, usually abruptly tapering to a rigid median nerve ca. 1 mm, about equaling disc-florets. Ray floret achenes tuberculate, striate, trigonous, curved, 1.5–1.75 mm, with a rim ca. 0.1 mm. Outer disc floret achenes slightly tuberculate and slightly striate, trigonous, (1.3–)1.5–1.9 mm, with an acute rim ca. 0.1 mm; inner disc floret achenes not or slightly tuberculate, usually slightly striate, turbinate, quadrangular, 1.6–2 mm, with an acute rim ca. 0.1(–0.2) mm.

*Distribution and habitat:* Balkan endemic growing in Greece, Bulgaria and North Macedonia [7]. In Greece, it is apparently restricted to the Rodopi mountain range, at the north-eastern part of the country. More widespread but rare and threatened in Bulgaria [31,32]. It grows in semi-shaded woodland edges and sometimes in rock fissures, at an elevation of 1000–1850 m. Young individuals consisting of rosette leaves usually present at flowering time.

*Notes:* Some plants located in the Frakto Virgin Forest (Mt Rodopi) deviate from the rest of the examined *A. macedonica* subsp. *orbelica* populations in their much wider, dark brown margins of the involucral bracts and the shallowly hemispherical, almost flat involucre. These specific plants were found ca. 400 m apart a population of *A. pindicola* Halácsy, whereas several other specimens of the typical *A. macedonica* subsp. *orbelica* have been collected in the wider area. They may represent hybrids between the two above-mentioned taxa as the involucre shape and the bract characters witness. Backcrosses with the parental species are also possible.

***Anthemis meteorica*** Hausskn. ex Nyman (1893: 125) (Figure 11)

*Lectotype (designated here):* Greece. Thessaliae: pr. mon. Meteora, Jun. 1885 (JE00006671!).

*Isolectotype:* Thessalia superior (JE00006670!). 

*Description:* Biennial or short-lived perennial, sometimes flowering in the first year, sparsely pubescent to pubescent with medifixed hairs, more or less densely glandular-punctate in all parts. Stem single or usually several, erect, (10–)15–35 cm long, branched, striate, often subquadrangular and reddish-brown, at least in the lower parts. Non-flowering shoots sometimes present at flowering. Leaves pinnatisect, the primary segments patent, usually pubescent; ultimate leaf lobes very narrow, (0.4–)0.6–0.7 mm wide, narrowly oblanceolate to linear, appearing almost cylindrical and acicular, with a sharp mucro up to 0.2 mm. Lower stem leaves 1.2–3 cm, 2-3-pinnatisect; upper leaves smaller, 1- to 2-pinnatisect. Peduncles (2–) 5–8 cm long, leafless, except for small, scale-like, entire leaves; capitula solitary. Involucre 8–9 mm wide, hemispherical, slightly pubescent to almost glabrous; involucral bracts yellowish-green, with a concolorous to slightly darker, prominent midvein, the margins usually pale; outer bracts 2–3 × 1–1.5(–1.8) mm, ovate to lanceolate, subacute to acute; inner bracts (3–)4–5 × 1–2 mm, lanceolate to oblanceolate, usually subacute. Ligules white, 7–10(–12) mm. Disc florets yellow, (2.3–)2.7–3 mm, lower 1–1.3(–1.5) mm part swollen at maturity, lobes 0.3 mm. Receptacle elongated hemispherical-cylindrical, obtuse to subacute, 3.5–6 × 2.5–3.5 mm; receptacular scales oblong-obovate, tapering into a rigid acumen 0.5–1 mm long, about equaling disc-florets. Ray floret achenes slightly tuberculate, slightly striate, trigonous, curved, 1.2–1.5 mm, with a rim ca. 0.1 mm. Outer disc floret achenes slightly tuberculate or smooth, slightly striate, subquadrangular, curved, 1.3–1.7 mm, with an acute rim ca. 0.1–0.2 mm; inner disc floret usually not tuberculate, slightly striate, achenes turbinate, quadrangular, 1.4–1.9 mm, with an acute rim ca. 0.1–0.2 mm.

*Distribution and habitat:* Balkan endemic, distributed in Greece, Albania and North Macedonia [7]. In Greece, it is known from its locus classicus in Meteora and a few additional localities in the east-central and north-central parts of the mainland (Figure 10). It grows in rather dry, open places, in meadows or scrub, at an elevation of 500–1500 m.

**Figure 12:**
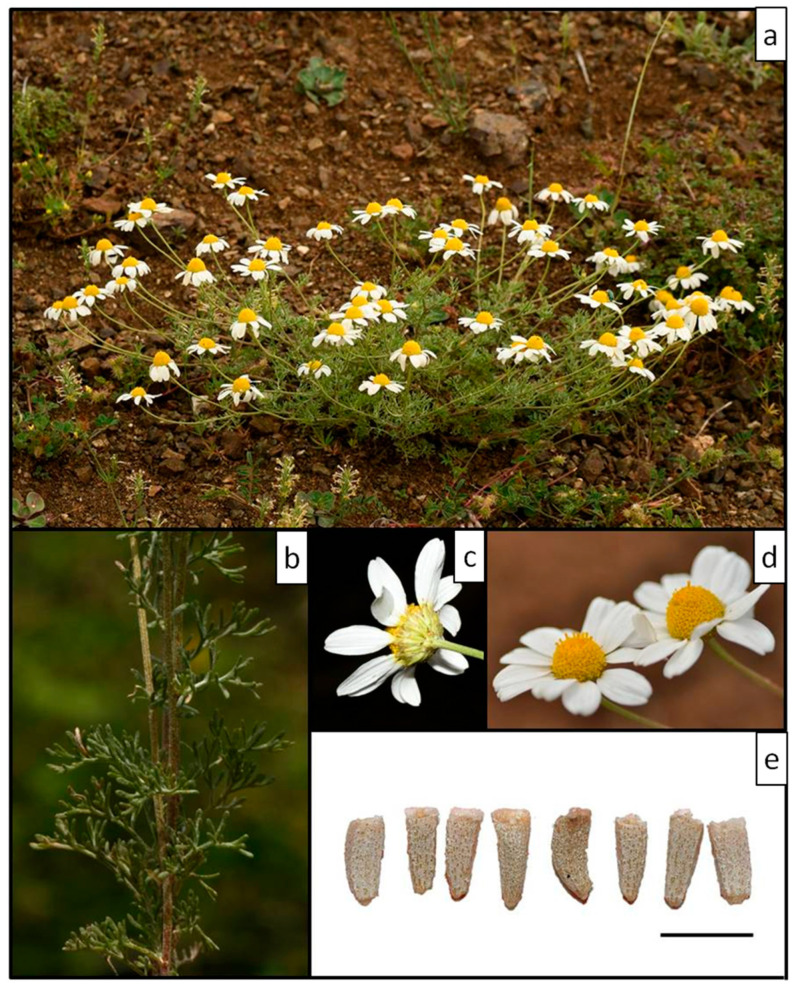
*Anthemis serpentinica* habit (**a**), leaves (**b**), involucre (**c**), capitula (**d**) and achenes (**e**). Scale bar = 2 mm.

***Anthemis serpentinica*** Goula & Constantinidis, ***sp. nova*** (Figure 11 and Figure 12)

*Type:* Greece. Nomos Fthiotidos, ca. 17 km NNW of Lamia town, road embankment with sparse *Juniperus* shrub, serpentine, 38°59 N/22°22 E, 763 m, 14 May 2018, *K. Goula 2464* (holotype, ATHU; isotype, B).

*Description:* Predominately biennial plant, pubescent to tomentose at least when young, with medifixed hairs, more or less densely glandular-punctate in all parts. Stems several, ascending to erect, 15–35 cm long, branched, striate, often subquadrangular and reddish-brown at least in the lower parts. Non-flowering shoots usually absent at flowering. At least some non-flowering rosette plants present together with flowering individuals in the field. Leaves pinnatisect, the primary segments patent, tomentose; ultimate leaf lobes very narrow, 0.5–0.7 mm wide, narrowly obovate, obtuse or with a minute mucro. Lower stem leaves 1.5–2.5 cm, 3-pinnatisect; upper leaves 2- to 3-pinnatisect. Peduncles (5–)7–11 cm long, leafless, except for a few small, scale-like, entire leaves; capitula solitary. Involucre 9–10 mm wide, hemispherical to obconical, tomentose when young, later slightly pubescent; involucral bracts yellowish-green, with a concolorous prominent midvein, the margins very thin, pale or light brown; both outer and inner bracts lanceolate with acute apex; outer bracts (1.5–)2–3 × 1.2–1.6 mm, inner bracts 4.5–5 × 1.5–2 mm. Ligules white, 10–13 mm. Disc florets yellow, 2.8–3.2 mm, lower 1.5–2 mm part swollen at maturity, lobes 0.4 mm. eceptacle elongated conical, sharply acute, 4.5–6.5 × 3–3.5(–4) mm; receptacular scales oblong-obovate, tapering into a rigid acumen ca. 0.5 mm long, about equaling disc-florets. Ray floret achenes more or less tuberculate, striate, trigonous, curved, 1.8–2 mm, with a rim ca. 0.1 mm. Disc floret achenes granulate or slightly tuberculate, 1.8–2.1 mm, with an acute rim, forming a short, usually oblique corona up to 0.2–0.3 mm; outer disc floret achenes usually subquadrangular, inner disc floret achenes turbinate, quadrangular.

*Distribution and habitat:* Endemic to Central Greece, where it has so far been located only in a few localities on the ultramafic substrate that forms a continuum from the western parts of Mt Othris area (eastern border) to the low hills around Mt Tamasio (Mt Katachloro) to the west. It grows on bare slopes, scree, and road embankments, together with the local serpentine endemic *Silene fabaria* subsp. *domokina* Greuter. Other plant species that were found growing at its locus classicus are *Convolvulus cantabrica* L., *Crucianella graeca* Boiss., *Echium italicum* L., *Melilotus neapolitanus* Ten., *Minuartia attica* (Boiss. & Spruner) Vierh. subsp. *attica*, *Onobrychis caput-galli* (L.) Lam. and *Thymus teucrioides* subsp. *candilicus* (Beauverd) Hartvig. Young individuals consisting of rosette leaves present at flowering time.

*Notes: Anthemis serpentinica* is the southernmost member of the *A. macedonica* group in Greece. Compared to its geographically closest relative, *A. meteorica*, of which the nearest population is located ca. 70 km to the north (Figure 10), the new species is larger in several of its parts, including the involucre width, ligule, disc floret, and achene length, although it has a similar stem height and leaf size with *A. meteorica*. Regarding the indumentum, it is certainly more densely pubescent than *A. macedonica* s.l. and *A. meteorica*. The populations examined (see Appendix A) present a morphological stability that we interpret as the result of speciation on serpentine rather, than serpentinomorphosis (see [33,34]). Seven additional obligate serpentine endemics are found in the same area [35]. Serpentine has long been considered as a driving force in plant evolution and speciation (e.g., [36]). Another interesting example of *Anthemis* speciation on serpentine is *A. rhodensis* Boiss.: both its subspecies, subsp. *rhodensis* and subsp. *pulvinalis* Rätzel & Ristow grow on the ultramafic substrate of Rodos Island [37].

***Anthemis thracica*** (Griseb.) Stoj. & Acht. (1948: 1150) (Figure 11)

*Basionym: A. montana β. thracica* Griseb. (1846: 2).

*Type:* Unknown country. In campis Thraciae, frequentissime in lapidosis pr. Ruskoi, *Grisebach 322* (holotype, GOET001036!).

*Synonyms: A. orientalis* subsp. *thracica* (Griseb.) Stoj. & Acht. (1937:515); *A. kitanovii* Thin (1980: 379); *A. macedonica* subsp. *thracica* (Griseb.) Oberpr. & Greuter (2003: 40).

*Description:* Predominantly perennial, sometimes flowering the first year, tomentose to tomentose-sericeous, with medifixed hairs, more or less densely glandular-punctate in all parts. Stems usually several, erect, 10–30 cm long, sparingly branched, striate. Leaf-rosette usually absent at flowering. Leaves pinnatisect, the primary segments patent, tomentose to tomentose-sericeous; ultimate leaf lobes very narrow, 0.5–0.8 mm wide, narrowly ovate to obovate, usually with a minute mucro. Lower stem leaves (1.2–)1.8–3(–4) cm, 2-pinnatisect; upper leaves smaller, (1-)2-pinnatisect. Peduncles (2–)3–10 cm long, leafless; capitula solitary. Involucre 7–9 mm wide, hemispherical to cupuliform, tomentose-sericeous; involucral bracts yellowish-green, with a concolorous, prominent midvein, the margins pale; outer bracts (1.5–)2–3 × (0.8–)1–1.5 mm, ovate to lanceolate, subacute; inner bracts 3–5 × (1–)1.3–2 mm, lanceolate to oblanceolate, subacute to acute. Ligules white, (5–)6–8 mm. Disc florets yellow, 2.2–2.3(–2.5) mm, lower 1–1.2 mm part swollen at maturity, lobes 0.2–0.3 mm. Receptacle short, hemispherical or conical, obtuse or acute, 2–4 × 2.5–4 mm; receptacular scales oblong-obovate, usually abruptly tapering into a rigid median nerve ca. 0.5 mm long, about equaling or slightly shorter than disc-florets. Ray floret achenes tuberculate, slightly striate, trigonous, curved, 1.2–1.5 mm, with a corona up to 0.3 mm. Outer disc florets achenes usually tuberculate, usually not striate, obconical or subquadrangular, curved, 1.5–2 mm, with a corona 0.4–0.7 mm; inner disc floret achenes granulate to tuberculate, usually not striate, subquadrangular, 1.5–2 mm, with a corona 0.4–0.5 mm long.

*Distribution and habitat:* Balkan endemic growing in Greece and Bulgaria [7]. Restricted to the north-eastern parts of the countries [31,32]. It grows mainly on dry hills, at an elevation of 90–700 m.

## 4. Materials and Methods

Plant specimens of *Anthemis macedonica* s.l. (including *A. meteorica*) from ATH, ATHU, TAU, TAUF, UPA were studied in detail. Digital images of plants preserved in B, BEOU, BM, BRNM, G, GOET, JE, K, LD, LY, PRC, W, WU were also studied, with an emphasis on the type material. The herbarium acronyms appearing above follow [38]. The protologues of all the representatives of the group were researched and studied. Moreover, the descriptions, nomenclature, and evaluation of the taxonomic relationships of the taxa were studied in both historic and recent literature [8,9,10,11,12,13,14,15,16,17,18,19,20,21]. Descriptive terminology follows Stearn [39]. Plant material from Northern and Central Greece (the natural distribution of the group) was investigated in the field during excursions in spring and summer of 2017–2020. Several voucher specimens were made for the morphometric work, ripe achenes were collected when possible, sown, and young plants were cultivated experimentally for both chromosome investigations (see below) and phenotypic observations. The morphological variation of the group was studied in recent and intact herbarium specimens prepared from various localities and deposited in ATHU. For *A. macedonica* subsp. *stribrnyi,* we checked the type but had no adequate material for further analyses.

The morphometric analysis was carried out based on the detailed measurements of 95 plants, corresponding to 26 populations, and covering the *Anthemis macedonica* group (*A. macedonica* subsp. *macedonica*, *A. macedonica* subsp. *thracica*, *A. orbelica*), *A. meteorica* from its locus classicus at Meteora (Central Greece) and nearby areas, as well as populations of a particular form growing on the serpentine areas of Central Greece that approached *A. macedonica* s.l. phenotypically (*Anthemis 1*). The analysis was carried out mostly on Greek samples. A total of 20 quantitative morphological characters were measured on the herbarium specimens and three ratios were calculated (Table 1). In addition, 19 qualitative characters were evaluated (Table 1). Many of the selected characters are among those considered as diagnostic for the members of the group and are mentioned as such in the literature. To explore the dominant patterns in the morphological variation of the examined specimens, the principal components analysis [40] was applied on the quantitative morphological characters by using the “princomp” function of the R package “stats” [41]. To explore the variability of both the quantitative and qualitative morphological characters, the factor analysis on mixed data (FAMD [42]) was used, a method that is applicable to a dataset containing both continuous and categorical variables. The FAMD was applied using the “FAMD” function of the “FactoMineR” [43] R package.

The karyological examinations were made in the root tips obtained from cultivated material. Mature achenes from eight populations collected from various localities were sown in pots at the facilities of the Department of Biology, National and Kapodistrian University of Athens. The seedlings were transferred in individual pots and root tips were collected several times. The protocol used for the pretreatment of the roots, the preparation of metaphase plates and the construction of idiograms is described in [44]. The total haploid length (THL, see [45]) was calculated to provide a chromosome size evaluation. The indices M_CA_ [27], CV_CL_/CV_CI_ [28] and A1/A2 [29] were used to estimate the intrachromosomal and interchromosomal asymmetry. All chromosome measurements and the evaluation of the asymmetry indices were conducted using the KaryoType software, ver. 2.0 [46].

## Figures and Tables

**Table 1:** Quantitative characters, ratios and qualitative characters used in the morphometric analysis of the *Anthemis macedonica* group. All measurements in mm.

**Quantitative Characters**			
MNB	Maximum number of stem branches at the lower part of stem	OBMW	Outer bracts margin width
BLL	Basal leaf length	IBL	Inner bracts length
DSLs	Number of divisions in stem leaves	IBW	Inner bracts width
ULLW	Ultimate leaf lobes width	IBMW	Inner bracts margin width
LML	Leaf mucro length	OACL	Outer disc floret achenes corona length
LL	Ligule length	OAL	Outer disc floret achenes length
IW	Involucre width	OAW	Outer disc floret achenes width
DFLL	Disc floret lobes length	IACL	Inner disc floret achenes corona length
OBL	Outer bracts length	IAL	Inner disc floret achenes length
OBW	Outer bracts width	IAW	Inner disc floret achenes width
**Ratios**			
SHLULSR	Total stem length/upper leafless stem	DFLTPLR	Total disc floret length/thickened part length
RLRWR	Receptecle length/receptacle width		
**Qualitative Characters**			
LG	Longevity	IP	Involucre pubescence
BRs	Stem branches	IS	Involucre shape
ULLS	Ultimate leaf lobe shape	RS	Receptacle shape
LP	Leaf pubescence	OAT	Outer disc floret achenes surface: tuberculation
OBS	Outer bracts shape	OAS	Outer disc floret achenes surface: striation
OBA	Outer bracts apex	OASH	Outer disc floret achenes shape
IBS	Inner bracts shape	IAT	Inner disc floret achenes surface: tuberculation
IBA	Inner bracts apex	IAS	Inner disc floret achenes surface: striation
BMC	Bracts margin colour	IASH	Inner disc floret achenes shape
SCS	Scale shape		

**Table 2:** Chromosome counts in populations of the *Anthemis macedonica* group, together with their karyotype formulas, total haploid length (THL) and asymmetry indices M_CA_ and CV_CL_.

Taxon	Origin	2*n*	Formula	THL	CV_CL_	M_CA_
*A. macedonica* subsp. *macedonica*	(a) Kastoria, (b) Thessaloniki	18	12m + 2sm + 4st^sat^	31.76–41.04	14.84–17.82	27.33–29.88
*A. macedonica* subsp. *thracica*	Evros	18	12m + 4sm + 2st^sat^	32.29–37.72	13.99–19.6	21.21–25.2
*A. meteorica*	(a) Grevena, (b) Trikala	18	12m + 4sm + 2st^sat^	31.19–37.47	7.84–21.65	22.46–26.81
*A. orbelica*	(a) Drama-Frakto, (b) Drama-Livaditis	18	12m + 2sm + 4st^sat^	37–49.16	11.05–17.89	21.64–27.02
*Anthemis 1*	Magnisia	18	12m + 2sm + 4st^sat^	31.58–39.18	16.23–19.72	24.02–30.61

**Table 3:** Comparison of morphological features among five taxa of the *Anthemis macedonica* group. For *A. macedonica* subsp. *stribrnyi,* only the type was seen. Uncommon characteristic values are in parentheses. All measurements in mm.

	*A. macedonica* subsp. *macedonica*	*A. macedonica* subsp. *orbelica*	*A. meteorica*	*A. serpentinica* (*Anthemis 1*)	*A. thracica*
**Lifespan**	Annual to biennial or short lived perennial	Biennial, sometimes flowering from the first year, or short lived perennial	Biennial, sometimes flowering from the first year, or short lived perennial	Predominately biennial	Predominantly perennial, sometimes flowering from the first year
**Individual leaf rosettes at flowering period**	Usually absent	Usually present	Absent	Present	Absent
**Leaf pubescence**	Sparsely pubescent to glabrous	Sparsely pubescent to subglabrous	Usually pubescent	Tomentose	Tomentose to tomentose-sericeous
**Involucre pubescence**	Glabrescent	Slightly pubescent to glabrescent	Slightly pubescent to almost glabrous	Pubescent to tomentose	Tomentose- sericeous
**Receptacle shape**	Shortly conical to hemispherical-cylindrical	Shortly conical to hemispherical-cylindrical	Elongated hemispherical-cylindrical	Elongated conical, apex sharply acute	Hemispherical to shortly conical
**Inner involucral bracts shape**	Oblanceolate to obovate, apex usually obtuse	Oblanceolate to obovate, apex obtuse to subacute	Lanceolate to oblanceolate, apex subacute	Lanceolate, apex acute	Lanceolate to oblanceolate, apex subacute to acute
**Involucral bracts margin**	Light to dark brown	Usually brown to dark brown	Not coloured	Very thin, light brown or pale	Not coloured
**Involucral bracts midvein**	Green, darker than rest of the bract	Green, much darker than rest of the bract	Concolorous or slightly darker than rest of the bract, prominent	Concolorous with the rest of the bract, prominent	Concolorous with the rest of the bract, prominent
**Disc florets thickened part length**	1–1.2	1–1.1	1–1.3 (–1.5)	1.5–2	1–1.2
**Disc florets length**	(2.4–) 2.5–2.8 (–3)	(2.3–) 2.5–2.8	(2.3–) 2.7–3	2.8–3.2	2.2–2.3 (–2.5)
**Achenes corona length**	0.1	0.1 (–0.2)	0.1–0.2	0.2–0.3	0.4–0.7

## Data Availability

See Appendix A.

## Appendix A. Examined Specimens

*The Populations Examined Karyologically Are Marked as [**chrom.**]*

***Anthemis macedonica*** subsp. ***macedonica***

**GREECE. Nomos Dramas:** 22 km from Paranestion along road to Zagradenia, rocky outcrop in deciduous forest, 350 m, 20/6/1988, *Strid et al. 27163* (ATH 59477); 1.5 km SW of Aidonokastro, 245 m, 24/8/2018, *Goula & Katsaros 2732* (ATHU); Dipotama, edges of *Quercus* forest, 610 m, 8/6/2019, *Charalampidou 521* (unpublished material); Potami, opening in *Quercus* forest, 410 m, 20/6/2019, *Charalampidou 780* (unpublished material); Mt Orvilos, along dirt road on the S slopes, 1069 m, 26/7/2020, *Goula & Katsaros 3162* (ATHU). **Nomos Chalkidikis:** Sarti, evergreen scrub with *Pinus halepensis* and *Erica manipuliflora*, 231 m, 4/5/2014, *Damianidis 1837* (TAUF); Sarti, evergreen scrub with *Pinus halepensis* and *Erica manipuliflora*, 475 m, 11/6/2014, *Damianidis 2471* (TAUF); Ibid., *Damianidis 2477* (TAUF). **Nomos Florinis:** Mt Varnous, E of the village of Agios Germanos, meadows, 1400 m, 28/6/1981, *Strid et al*. *18266* (ATH 59480; LD 1212509); Mt Varnountas, 2.4 km SE of Pisoderi, 1523 m, 22/6/2020, *Goula 3144* (ATHU); Ibid., 10/8/2020, *Goula & Katsaros 3171* (ATHU); Mt Varnountas, N of Vigla, along dirt road to wind farm, 1780 m, 10/8/2020, *Goula & Katsaros 3174* (ATHU). **Nomos Imathias:** S of the village Kastanea, in *Fagus* forest, sandy-clayey humus soil, 1150 m, 26/6/1971, *E. Stamatiadou 12992* (ATH 16919); Mt Vermio, 4 km SW of Kastania, 1315 m, 16/7/2018, *Goula 2718* (ATHU). **Nomos Kastorias:** Mt Vitsi, sandy road embankment, 1236 m, 22/7/2017, *Goula et al. 2204* (ATHU); Mt Vitsi, opening in *Fagus sylvatica* forest, 1477 m, 10/8/2019, *Goula & Katsaros 3067* (ATHU) [**chrom.**]. **Nomos Kavalas:** Mt Pangeo, rocky opening in *Fagus* forest, 1301 m, 4/6/2019, *Goula et al. 2900* (ATHU); Ibid., 26/7/2019, *Goula & Katsaros 3003* (ATHU). **Nomos Kilkis:** 1.7 km SE of Akropotamia, dry meadow and abandoned field, 291 m, 31/5/2019, *Goula 2859* (ATHU); 3.6 km SE of Pontokerasia, opening in *Quercus* forest, 518 m, *Goula 2868* (ATHU); 750 m NE of Kokkinia, opening in deciduous forest, 329 m, 31/5/2019, *Goula 2871* (ATHU). Nomos Kozanis: Near the village of Metaxas, grassland on schistose hills, 1000-1050 m, 2/6/2001, *Strid et al. 52703* (ATH 59475; LD 1212389); 1.2 km SSE of Driovouno, rocky road embankment and dry meadow, 756 m, 6/6/2019, *Goula & Proios 2912* (ATHU); 3.4 km NE of Sisani, road embankment in *Quercus* forest, 924 m, 6/6/2019, *Goula & Proios 2915* (ATHU). **Nomos Pierias:** Mt Olympus, N side, along forest road on E side of Papa Rema ravine, *Pinus nigra* woodland and limestone rocks, 920-980 m, 10/6/1976, *Strid & Kjellsson 11324* (ATH 59474). **Nomos Serron:** Montes Vrondous, silva Lailia, a refugio orientem versus, in scansilibus graminosis rupium graniticarum, 1500-1600 m, 17/6/1973, *W. Greuter 11233* (ATH 29513). **Nomos Thessalonikis:** In m. Korthiati Macedoniae, 20/7/1857, *Oprhanidis s.n.* (LY0017447); Legi in reg. super. montis Corfiati, Macedoniae, 20/7/1857, *Orphanidis 3614* (G00764108); Ibid. ATHU; Montes Karadagh inter Thessaloniki et Serrai, 50-60 km viae publicae, ca. 500 m, 8/6/1936, *K.H. & F. Rechinger 9210* (LD 1993560). Inter Lahanas et Evangelistria, in pratis et dumetis caducifoliis, 650 m, 5/6/1973, *W. Greuter 11083* (ATH 29505); 4 km NE of Dorkada, fallow field, 578 m, 31/5/2019, *Goula 2857* (ATHU); 2.8 km NE of Dorkada, in mixed *Pinus*-deciduous forest, 512 m, 31/5/2019, *Goula 2858* (ATHU); Ibid., 26/7/2019, *Goula & Katsaros 3005* (ATHU) [**chrom.**].

***Anthemis macedonica*** **subsp.** ***orbelica***

**GREECE. Nomos Dramas:** SW of the village Katafito, sandy, clayey ground, 850 m, 23/7/1977, *E. Stamatiadou 20102* (ATH 39848); In ditione Elatia (Kara Dere), a pago Skaloti, 10 km septemtriones versus, 1550 m, 19/8/1978, *Greuter 16573* (B 10 1149346, UPA 8647); Mt Rodopi, N of the forest station Zagradenia, meadow in *Picea* forest, in rocky places, 1800-1900 m, 9/8/1979, *Strid et al. 16477* (ATH 60763); Rhodopengebiet N von Drama, Umgebung der Forststation Zagradenia, 6 km N von Zagradenia Frakto, Straßenböschung, ca. 1540 m, 13/6/1987, *Oberprieler 2992* (B 10 0311198); W Rodopi, Frakto Virgin Forest (Agriogido), grassland and clearings of *Picea* forest, 1820 m, 13/7/1993, *Eleftheriadou 2182* (TAUF); Ibid., grassland, 1850 m, 14/7/1993, *Eleftheriadou 2226* (TAUF); W Rodopi, Frakto Virgin Forest (Kapsalaki), margins of *Picea* forest, 1820 m, 9/9/1993, *Eleftheriadou 2463* (TAUF); W Rodopi, Frakto Virgin Forest (between Likolakka and the Bulgarian border), clearings in *Picea* forest, 1850 m, 10/9/1993, *Eleftheriadou 2488* (TAUF); W Rodopi, Frakto Virgin Forest (Agriogido), grassland, 1750 m, 23/6/1994, *Eleftheriadou 2709* (TAUF); Ibid., 1650 m, 19/7/1994, *Eleftheriadou 2836* (TAUF); Frakto virgin forest near the Bulgarian border, meadows in opening of *Picea abies* forest, schist, 1750-1900 m, 19/7/1997, *Strid et al. 44447* (ATH 59476, LD 1215388); Mt Rodopi, Frakto area, rocky meadow in opening of *Pinus sylvestris-Picea abies* forest, 1380 m, 13/8/2017, *Goula & Katsaros 2258* (ATHU); Ibid., *Goula & Katsaros 2259* (ATHU); Mt Rodopi, Frakto area, mixed *Picea, Abies* and *Fagus* forest, close to the Bulgarian border, 1728 m, 25/7/2020, *Goula et al. 3157* (ATHU) [**chrom.**]; Mt Rodopi, Elatia area, in *Picea abies-Pinus sylvestris* forest, 1340 m, 14/7/2017, *Goula & Katsaros 2267* (ATHU); Mt Rodopi, Elatia area, in *Picea abies* forest, 1425 m, 13/8/2017, *Goula & Katsaros 2269* (ATHU); Ibid., 1551 m, 14/7/2017, *Goula & Katsaros 2270* (ATHU); Ibid., 1545 m, 14/7/2017, *Goula & Katsaros 2271* (ATHU); Ibid., 1538 m, 14/7/2017, *Goula & Katsaros 2272* (ATHU); Mt Rodopi, Elatia area, in *Picea abies-Pinus sylvestris* forest, 1383 m, 25/7/2019, *Goula & Katsaros 2997* (ATHU); Ibid., 25/7/2019, *Goula & Katsaros 3000* (ATHU); Mt Rodopi, Elatia area, in *Picea abies* forest, 1554 m, 25/7/2019, *Goula & Katsaros 3001* (ATHU); Mt Rodopi, Livaditis area, in *Fagus sylvatica* forest, 1161 m, 17/8/2017, *Goula & Katsaros 2283* (ATHU) [**chrom.**]; Ibid., 20/7/2019, *Goula & Katsaros 2962* (ATHU); Mt Rodopi, Simida Forest, in mixed *Pinus-Abies* forest with *Betula pendula*, 1400 m, 22/7/2019, *Goula & Katsaros 2967* (ATHU); Mt Rodopi, Simida Forest, edges of forest road, 1117 m, 22/7/2019, *Goula & Katsaros 2968A* (ATHU). **BULGARIA.** In pratis ac dumosis saxosis que m. Rilo, 8/1882, *Pančić s.n.* (BEOU 9925); M. Rilo (BEOU 9939); M. Rilo supra coenobium [], 7/1887, *Bornmuller s.n.* (WU 0036349); Ostrec in mm. Rhodope Bulgariae, 2/8/1897, *Formánek 1/406* (BRNM 15362/35); In graminosis valle [] supra v. Rilo, 1889, *Velenovský s.n.* (SOM 78406); Trojan Balkan, 5/1899, *Urumoff 474* (PRC 451226), var. *lucida*; Montes Rila-Planina, in declivibus australibus supra [], substr. silic., 300 m, 4-5/8/1930, *Rechinger s.n.* (WU 0036348). **UNKNOWN COUNTRY**. In herbidis saxosis reg. mont. mt. Rhodope, 7/1906, *Adamovic s.n.* (WU 0036407) [as *A. meteorica*].

***Anthemis macedonica*** subsp. ***stribrnyi***

**BULGARIA.** In m. Rhodope supra [Lavorova], 4/8/1895, *Stribrny s.n.* (K000901776).

***Anthemis meteorica***

**GREECE. Nomos Florinis:** Mt Varnountas, 1.5 km SSE of Agios Germanos, dry meadow, 1409 m, 16/7/2018, *Goula & Sakellarakis 2714* (ATHU). **Nomos Grevenon:** Mt Kamvounia, 1 km NNW of Deskati, dry meadow, 12/7/2018, *Goula & Polymenakos 2672* (ATHU); Mt Kamvounia, 2.5 km N of Deskati, 1143 m, 12/7/2018, *Goula & Polymenakos 2674* (ATHU) [**chrom.**]; Mt Kamvounia, 6 km N of Deskati, 1375 m, 12/7/2018, *Goula & Polymenakos 2685* (ATHU). **Nomos Larisis:** Between Loutro and Akri villages, 944 m, 12/7/2018, *Goula & Polymenakos 2689* (ATHU); 4 km SW of Spilia, 627 m, 28/5/2019, *Goula 2837* (ATHU). **Nomos Trikalon:** Meteora, *Haussknecht s.n.* (JE00006670); Thessaliae pr. mon. Meteora, 6/1885, *Haussknecht s.n.* (JE0006671); Pindus Tymphaeus. In apricis supra Kalabaka ad monasteria Meteora, 7/1885, *Haussknecht s.n.* (WU0035422); In saxos. pr. monast. Meteora supra Kalabaka, 14/7/1885, *Haussknecht s.n.* (BM000932599); Ibid., *Haussknecht s.n.* (W 1880-0000524); Pr. monast. Meteora in saxosis, 14/7/1885, *Haussknecht s.n.* (K000901775); Meteora, road embankment, 498 m, 11/5/2018, *Goula 2418* (ATHU); Ibid., 11/7/2018, *Goula & Polymenakos 2563* (ATHU) [**chrom.**].

***Anthemis serpentinica***

**GREECE. Nomos Fthiotidos:** 18 km NNW of Lamia, rocky road embankment with sparse vegetation, serpentine, 751 m, 11/5/2018, *Goula 2417* (ATHU); 20 km NNW of Lamia, road embankment, serpentine, 767 m, 14/5/2018, *Goula 2464* (ATHU, B); Ibid., 9/6/2018, *Goula 2591* (ATHU); 3.5 km S of Ekara, ophiolithic substrate, *Goula & Katsaros obs*. **Nomos Karditsis:** C. 3.8 km S of Kedros village, along road to Loutra Smokovou, ophiolithic substrate, 220-330 m, 23/5/1998, *Th. Constantinidis & A. Iliadis 7775* (ATHU). **Nomos Magnisias:** 3.8 km SW of Anavra, serpentine, 820 m, 28/5/2019, *Goula 2830* (ATHU); Ibid., 11/8/2019, *Goula & Katsaros 3069B* (ATHU) [**chrom.**].

***Anthemis thracica***

**GREECE: Nomos Evrou:** Mt Boukate-Dagh, NW of the abandoned village Pessani, damp, gravelly places in *Quercus* woodland, 650-700 m, 20/5/1972, *E. Stamatiadou 15243* (ATH 25160); In montibus Boukate dag a pago Esimi c. 10 km septemtriones versus. In pascuis petrosis inter dumulos juniperi, solo calcareo, 750 m, 13/7/1978, *Greuter 15942* (UPA 8646); Area called Pessani, rocky serpentine outcrop surrounded by mixed deciduous woodland, 400 m, 6/6/2006, *Strid et al. 53081* (LD 1423678); Between areas called Pessani and Tris Vrises, meadows in opening of deciduous oak woodland, schist, 550 m, 8/6/2001, *Strid et al. 53201* (ATH 59478; LD 1214968); Rocky area W-NW of the ecotourist station, fissures and shallow soil on rocks, 4/6/2006, *Snogerup & Lassen 21775* (LD 1121779); 3 km SE of Leptokaria village, opening in deciduous oak woodland, 673 m, 2/6/2018, *Goula 2556* (ATHU); Kapsalos summit, in mixed deciduous forest, 588 m, 3/6/2018, *Goula 2557* (ATHU); Kapsalos summit, dry meadow in rocky area, 558 m, 3/6/2018, *Goula 2561* (ATHU); Dadia, near the Information Center of Dadia National Park, rocky area, 98 m, 3/6/2018, *Goula 2562* (ATHU); Area of Pessani, 322 m, 3/6/2018, *Goula 2564* (ATHU); SSW of Metaxades village, 247 m, 4/6/2018, *Goula 2574* (ATHU); 2 km SW of Mega Derio, dry meadow, schist, 486 m, 4/6/2018, *Goula 2577* (ATHU) [**chrom.**]; Mt Sapka, 664 m, 4/6/2018, *Goula 2579* (ATHU). **Nomos Rodopis:** In monte Karlik-Dagh prope Komotini, in declivibus australibus saxosis reg. Guere. substr. silic., c. 600 m, 2/7/1936, *K.H. & F. Rechinger 10406* (LD 1985243); Mt Papikio, artificial forest with *Pinus* and *Acacia*, 632 m, 23/7/2019, *Goula & Katsaros 2989* (ATHU); Ibid., *Goula & Katsaros 2990* (ATHU). **UNKNOWN COUNTRY.** In campis Thraciae, frequentissime in lapidosis pr. Ruskoi, *Grisebach 322* (GOET001036).

**Possible hybrids:**

***Anthemis macedonica*** subsp. ***macedonica* × *A. meteorica***

**GREECE. Nomos Pierias:** Mt Olimbos, along dirt road on the north side, 280 m, 10/6/2020, *Goula 3119* (ATHU).

***Anthemis macedonica*** subsp. ***orbelica* × *A. pindicola***

**GREECE. Nomos Dramas:** Mt Rodopi, Frakto area, mixed *Picea, Abies* and *Fagus* forest, close to the Bulgarian border, 1728 m, 25/7/2020, *Goula et al*. *3157A* (ATHU).
